# Skepticism towards the Swedish vision zero for suicide: interviews with 12 psychiatrists

**DOI:** 10.1186/s12910-018-0265-6

**Published:** 2018-04-10

**Authors:** Petter Karlsson, Gert Helgesson, David Titelman, Manne Sjöstrand, Niklas Juth

**Affiliations:** 1 Department of Learning, Informatics, Management and Ethics, Karolinska institutet, Tomtebodavägen 18A, 171 77 Stockholm, Sweden; 2 National Centre for Suicide Research and Prevention of Mental Ill-Health, Karolinska institutet, Granits väg 4, 171 77 Stockholm, Sweden

**Keywords:** Suicide, Prevention, Bioethics, Public health, Goals, Psychiatry

## Abstract

**Background:**

The main causes of suicide and how suicide could and should be prevented are ongoing controversies in the scientific literature as well as in public media. In the bill on public health from 2008 (Prop 2007/08:110), the Swedish Parliament adopted an overarching “Vision Zero for Suicide” (VZ) and nine strategies for suicide prevention. However, how the VZ should be interpreted in healthcare is unclear. The VZ has been criticized both from a philosophical perspective and against the background of clinical experience and alleged empirical claims regarding the consequences of regulating suicide prevention. This study is part of a larger research project in medical ethics with the overarching aim to explore whether the VZ is ethically justifiable. The aim is to enrich the normative discussion by investigating empirically how the VZ is perceived in healthcare.

**Methods:**

Interviews based on a semi-structured interview guide were performed with 12 Swedish psychiatrists. The interviews were analysed with descriptive qualitative content analysis aiming for identifying perceptions of the Vision Zero for Suicide as well as arguments for and against it.

**Results:**

Though most of the participants mentioned at least some potential benefit of the Vision Zero for Suicide, the overall impression was a predominant skepticism. Some participants focused on why they consider the VZ to be unachievable, while others focused more on its potential consequences and normative implications.

**Conclusions:**

The VZ was perceived to be impossible to realize, nonconstructive or potentially counterproductive, and undesirable because of potential conflicts with other values and interests of patients as well as the general public. There were also important notions of the VZ having negative consequences for the working conditions of psychiatrists in Sweden, in increasing their work-related anxiety and thwarting the patient-physician relationship.

**Electronic supplementary material:**

The online version of this article (10.1186/s12910-018-0265-6) contains supplementary material, which is available to authorized users.

## Background

Suicide is a serious public health problem worldwide [1, 2]. Globally, approximately 800,000 people die by suicide each year and it is the second leading cause of death among those aged 15–29 years [3]. In Sweden, approximately 1500 people complete suicide every year, and it is the most common cause of death in males aged 15–44 years [2, 4, 5].

Strategies for suicide prevention exist in most countries. The WHO presents a goal of reducing the global suicide rate by 10% before 2020 but also encourages its member states to adopt more ambitious goals [1]. The WHO argues that suicide prevention is imperative, that every suicide is a tragedy and that “every single life lost to suicide is one too many”. To the authors’ knowledge, Sweden is the only country with an official goal of reducing the suicide rate to zero. This is nevertheless of international relevance, since other countries or healthcare systems may suggest, or have already suggested, similar goals [6, 7].

In the bill on public health from 2008 (Prop 2007/08:110) [8], the Swedish Parliament adopted an overarching “Vision Zero for Suicide” (VZ) and nine strategies for achieving suicide prevention. The overarching vision states: “No person should find him- or herself in a situation in which they experience that the only solution is suicide. The government’s goal is that no person should take his or her own life”. The nine strategies are directed both towards the population and towards individuals. The three strategies that directly concern healthcare are labeled [1] “Medical, psychological and psychosocial efforts,” [2] “To increase the competence of personnel and other key actors in healthcare and care of persons with suicide related problems,” and [3] “To analyse critical incidents [of deaths by suicide in health service contexts] in *Lex Maria* reports”. The *Lex Maria* legislation concerns the healthcare provider’s responsibility to investigate incidents that have or could have resulted in injuries, in order to improve patient safety [9–11].

The main causes of suicide and how suicide could and should be prevented is an ongoing controversy in the scientific literature as well as in public media [12–19]. Contemporary scientific theories of the causes of suicide take medical, psychological and sociological aspects into account [20]. Epidemiological research up to this point indicates a high incidence of mental illness or drug abuse among persons completing suicide [21, 22]. However, the causes of suicide are complex and it is not established, nor can it be assumed, that mental illness or drug abuse are necessary causal factors that explain all suicides.

It is unclear how the VZ should be interpreted in healthcare. On the one hand, it is explicitly stated in the VZ that suicide prevention in Sweden should be based on a systematic approach taking all aspects of people’s social and physical environment into account [8]. Furthermore, the nine strategies to a great extent focus on areas outside of healthcare. On the other hand, empirical studies underscore the importance of psychiatric illness as a cause of suicide [1, 21, 22]. Moreover, regulations from the Swedish National Board of Health and Welfare introduced in connection to the VZ recommended mandatory inquiries of all suicides that occur in relation to healthcare in accordance with the *Lex Maria* legislation, which originally concerns incidents due to medical errors [11]. These regulations had the purpose of improving suicide prevention by learning from medical errors, but could also be interpreted to imply that every suicide in relation to healthcare in fact *is*, at least in part, the result of a medical error. The regulations have since been changed and it is at present up to healthcare to inquire if a suicide is to be considered the result of a medical error or not, and if so, it is to be inquired in accordance with the Lex Maria legislation [9, 10]. It is still the case however, that the VZ states that all instances of suicide in relation to healthcare should be investigated by the Swedish National Board of Health and Welfare. This seems to imply that the VZ includes an obligation for healthcare to prevent all suicides.

The VZ has been criticized both from a philosophical perspective and against the background of clinical experiences and alleged empirical claims regarding the consequences of regulating suicide prevention. For instance, it has been claimed about VZ that: (1) it is unachievable and therefore place an unreasonable burden on psychiatry [12]; (2) it hinders an open debate on legal euthanasia and physician-assisted suicide [13, 23]; (3) it may lead to an extensive use of paternalistic coercive measures in healthcare [13, 23, 24]; (4) it is misguided because some suicides, so called rational suicides, should not be prevented [23]; (5) it may be counterproductive [23].

This study is part of a larger research project in medical ethics with the overarching aim to explore whether the Swedish Vision Zero for Suicide is ethically justifiable. The aim of this study is to enrich the normative discussion of the VZ by investigating empirically how it is perceived in healthcare. Further, if policies are to be implemented in practice, it is important to find out what those who are supposed to implement them think about them. For instance, if there is a general scepticism towards the policy in question, this may undermine the feasibility of implementing it. For practical reasons the main focus of this study was set on psychiatry. In Sweden, psychiatrists frequently have a consulting role when suicidality is an issue in the somatic areas of healthcare. A focus on psychiatry could therefore be expected to cover problems relating to suicidality also when they appear in the somatic areas of healthcare, and the study was thus thought to concern healthcare as a whole. The aim of this study was to answer the following questions:

1. How is the Vision Zero for Suicide perceived by Swedish psychiatrists?
2. What arguments for and against the Vision Zero for Suicide occur amongst psychiatrists?
3. Do Swedish psychiatrists perceive that the Vision Zero for Suicide has influenced the clinical work in healthcare and, in that case, in what ways?

## Method

### Participants and study design

Interviews based on a semi-structured interview guide [see Additional file 1] were performed with 12 psychiatrists. The inclusion criteria for participation in the study were: (1) being a specialist or a resident in psychiatry and (2) having experience of working with suicidal patients. The aim was to obtain as rich qualitative data as possible. Thus, participants who differed in age, experience, area of expertise and sub-speciality were sought for. Participants were recruited continuously during the project by a mix of deliberative sampling and chain referral [25–28]. The number of participants was not set from the beginning but was decided depending on whether further interviews made any qualitative contributions to the already gathered material, weighed against a practical wish to limit the sample size. The final sample included five male and seven female psychiatrists, all employed in the Greater Stockholm area at the time of the interview. In total, the participants were working with both in-patient and out-patient care as well as with teaching, within general psychiatry as well as with focus on patients with affective disorders, neuropsychiatric disorders, personality disorders, or psychotic disorders. The participants’ clinical experience varied from current residency in psychiatry to having worked as a psychiatrist for 25 years.

The participants were initially contacted by email and if they were willing to participate they obtained a document with information about the design and purpose of the study, including that participation was voluntary and that consent could be withdrawn at any time during the course of the study. Verbal informed consent to participate was then obtained from each participant and documented in recording at the time of the interviews, which is sufficient according to the Swedish law. The verbal informed consent again followed information that participation was voluntary and that consent could be withdrawn at any time during the course of the study. The study was approved by the Regional Ethical Review Board in Stockholm (Dnr 2015/270–31/5).

### Semi-structured interviews

All interviews were conducted by the first author (PK). The interviews were semi-structured and based on five main questions with the possibility of adding qualifying follow-up questions, depending on the course of the interview [25, 28–30]. The interviews included questions about the participants’ experiences and perceptions of the Vision Zero for Suicide (VZ), as well as questions about their view on rational suicides [see Additional file 1]. The aim was to get answers to each question, but also to allow the participants to speak as freely as possible about them [25, 29, 30]. Hence, the participants were encouraged to elaborate on the questions or on their initial responses to them. The interviewer strived not to interrupt the participant while speaking, not to ask leading questions, and to allow silences [25, 29, 30]. There were a large number of follow-up questions, and topics were also covered that are not listed in the interview guide. The interviews were approximately between 45 and 75 min long. The interviews were recorded with a digital voice recorder and transcribed verbatim by the first author.

### Data analysis

The analysis was initially performed by the first author. The first author’s preunderstanding is based on his professional experience as a physician as well as being a PhD-student in medical ethics. The co-authors contributed to the analysis from their respective vantage point as philosopher, clinician, or suicide researcher.

The interviews were analysed with descriptive qualitative content analysis [27, 31]. The terminology suggested by Graneheim & Lundman was used: ‘subcategories’ refer to the manifest content of the interviews and ‘themes’ to the meaning running through the subcategories on a higher level of abstraction. The analysis was performed inductively, without pre-set categories or in relation to any theoretical framework [27, 31]. Though, with respect to the aim of the study, the analysis was focused on identifying perceptions of the VZ as well as arguments for and against it. In this sense the analysis was purposive.

Initially each participant’s transcribed interview was read through to get an overall impression of its content. Next, meaningful units were identified, i.e. words, sentences and paragraphs expressing a meaning. Thereafter, meaningful units similar in meaning were given a code, e.g. “There are no rational suicides”. Next, subcategories were created by sorting codes similar in meaning into the same subcategory. Further condensation of subcategories was done by arranging and re-arranging them into broader categories. Finally, categories were refined and sorted into overriding themes. The three levels subcategories, categories and themes thus represent increasing abstraction and condensation of information. Accordingly, the themes represent the highest level of abstraction and are the final product of the analysis.

The interest in finding perceptions of and arguments for or against the VZ rests upon the overarching aim of answering normative questions about it. This interest has steered the analysis of the interviews. Accordingly, overlapping categorization was considered justified if a reduction of subcategories or categories would have led to a loss of information that could be of potential normative importance.

## Results

The analysis of the 12 interviews yielded 26 distinct subcategories, 10 higher level categories, and four themes. The results are presented in Table 1 as well as in the text to follow. When subcategories are exemplified by more than one quote, in order to clarify their meaning, this does not imply greater importance.

**Table 1:** Results of content analysis of interviews with psychiatrists on the Vision Zero for Suicide

Subcategories	Categories	Themes
- Reach for the stars	Benefits in healthcare	Benefits of the VZ
- Increased awareness of suicidality in healthcare		
- A good way of supplying resources to suicide prevention		
- A positive signal from the authorities		
- Decreased shame and increased empathy for suicidal persons	Benefits for the general public	
- Increased public awareness of suicide		
- Insufficient medical knowledge to treat and prognosticate	Insufficient knowledge	The VZ is unachievable
- Insufficient knowledge for predicting suicide		
- Insufficient resources to prevent all suicides	Insufficient resources	
- Insufficient legal means for compulsory care	Limitations of compulsory care	
- Compulsory care is sometimes counterproductive		
- Misdirected focus at the expense of appropriate measures	Wrong focus	The VZ is nonconstructive
- Threat of suicide as an instrument of power		
- Decreased inclination to seek help or share problems		
- The VZ supports an unjustified difference between psychiatry and somatic care	Negative consequences for psychiatry	
- The VZ may have a deterring effect on physicians		
- The VZ may amplify contradictory tasks		
- The VZ may augment the anxiety among personnel		
- The VZ may contribute to false views of psychiatric illness among the public	Wrong message	
- The VZ may contribute to false hopes on healthcare among the public		
- The VZ contributes to medicalization of normal psychology		
- Thoughts and plans of suicide as comfort and hope		
- Conflicting values with regard to the patient	Some suicides should not be prevented because the moral cost is too high	The VZ is not desirable
- Conflicting values with regard to others		
- Rational suicide	Certain rational suicides should not be prevented	
- Assisted suicide		

### Benefits of the VZ

This theme consists of six subcategories sorted under two categories, and concerns what the participants perceived to be potential benefits from the Vision Zero for Suicide (VZ).

#### Benefits in healthcare

This category represents potential benefits from the VZ for suicide prevention in healthcare.

##### Reach for the stars

Two participants explicitly considered goals beyond one’s reach to be a constructive way of achieving desired results. Several other participants considered utopian goals to be mainly nonconstructive.

*I believe in the idea that, that you aim for the stars to reach the treetops in almost all respects. I think it’s good to have very high ambitions, if you at the same time can live with the idea that we may not reach all the way.*

##### Increased awareness of suicidality in healthcare

Several participants expressed that the VZ may lead to increased awareness of suicidality among healthcare staff, which potentially could prevent suicides.

*What is positive with the whole thing is that you, you constantly update this question of suicide and, maybe that you think about, you consider this question also in situations where you maybe wouldn’t otherwise.*

##### A good way of supplying resources to suicide prevention

Some participants expressed that the VZ may be a good way of directing resources to suicide prevention by drawing attention to the problem of suicide.

*I mean it’s a good way to supply resources to it, it illustrates a problem and […] everything that pushes things in that direction is positive.*

##### A positive signal from the authorities

Several participants expressed that the VZ is a positive signal from the authorities, and that it leads to suicide being taken seriously and as a problem in which the authorities are willing to invest.

*We signal that no one should be left to take his/her own life in a good country. It might signal that we’re willing to invest in this, that we take it seriously. It may of course be morally supportive for personnel in psychiatry and supportive for patients.*

#### Benefits for the general public

This category represents different ways in which the VZ may have benefits for the public.

##### Decreased shame and increased empathy for suicidal persons

Some participants expressed that the VZ may lead to decreased shame connected to suicide and facilitate talking about suicide in general, or increase people’s empathy with suicidal persons.

*So in this way it’s an advantage that you identify this as a problem and also something that we’re, that we’re able to talk about. That you don’t, just not talk about suicide. That it’s too shameful or taboo to even bring up.*

##### Increased public awareness of suicide

Some participants expressed that the VZ may enhance public awareness of suicidality, which in turn may prevent suicides.

*If it leads to knowledge. If it leads to the average citizen daring to ask the right questions when they’re worried about someone. That schools will take notice of this. If the vision leads to this, then I think it’s good.*

### The VZ is unachievable

This theme consists of five subcategories sorted under three categories and concerns what the participants perceive as reasons why the VZ is unachievable.

#### Insufficient knowledge

This category represents different ways in which a lack of knowledge is thought to be one of the problems with the VZ.

##### Insufficient medical knowledge to treat and prognosticate

Many participants expressed that one problem with the VZ is that current psychiatry has a limited ability to predict the course of illness, treatment outcomes, prognosis, and optimal choice of treatment for individual patients. This limitation, including the inability to ease the suffering of certain patients, was seen as a practical reason why the VZ is unachievable and as an argument against it.

*Sometimes it feels more like you carry out something reminding of witchcraft. In this situation you ought to perform certain rites in a certain way but you know that you have no chance whatsoever to take the proper measures.*

*They’ve tried every medication in the book, they’ve tried every therapy. They still get serious depressions. And I’m powerless and the patient is suffering. I mean sometimes it’s malignant illness that we really can’t handle today with the available science and current knowledge, unfortunately.*

##### Insufficient knowledge for predicting suicide

All of the participants expressed that one reason why the VZ is unachievable is a vast lack of evidence on how to assess suicide risk. The majority of participants expressed this notion in connection with the *Lex Maria legislation*, as it was written at the time of the interviews, which they perceived as being unreasonable. Further, they emphasized that statistically, suicides will occur also in what are labeled low-risk groups of patients.

*I think it’s very unfair if you’ve been in this situation where you have made a thorough assessment of your patient and decided that OK, you can be on leave during the weekend, at home with your family, and your dad will pick you up and so on. And then if you learn that she committed suicide, then it’s automatically our fault.*

*Because if you send people home who have a low risk of committing suicide, sooner or later for purely mathematical, statistical reasons, it will happen occasionally anyway.*

#### Insufficient resources

This category represents how the participants regard insufficient financial resources as one of the problems with the VZ.

##### Insufficient resources to prevent all suicides

A majority of the participants expressed that a lack of resources in psychiatry is a major reason why the VZ is unachievable.

*But I also think that this is a little offensive. If you work in the middle of it all, like I do, and experience that we are barely covered [economically] year after year after year. And then they say that we are going to add a little Vision Zero here. I don’t give a damn about the Vision Zero, give us money for more wards, [and] better healthcare.*

#### Limitations of compulsory care

This category represents different ways in which the participants perceive compulsory care and how it is regulated to be one of the problems with the VZ.

##### Insufficient legal means for compulsory care

Many participants expressed that even if it would be desirable to commit more people to inpatient care in order to prevent suicide, this would not be possible with the current legislation on compulsory psychiatric care.

*What happens then, if you hospitalize people – everybody would not want that obviously – you would need to hospitalize a lot of people against their will, and the current legislation does not allow that.*

#### Compulsory care is sometimes counterproductive

Many participants expressed that compulsory care is the only way for the physician to feel fairly certain that a patient will not attempt suicide notwithstanding that it is sometimes detrimental to the wellbeing and treatment of the patient and may even increase the risk of suicide in a longer time perspective, rendering it potentially counterproductive with respect to its own purpose.

*And an alternative maybe would’ve been to open the door and then she maybe would’ve run out and screamed for a bit and then she probably wouldn’t have taken her own life you see. It becomes, it becomes like a fish-hook in your hand, the more you try to fix it, the worse it gets.*

### The VZ is nonconstructive

This theme consists of 11 subcategories sorted under three categories and concerns what the participants perceive to be potentially negative consequences of the VZ.

#### Wrong focus

This category represents different ways in which the participants consider the VZ to misdirect the focus of healthcare.

##### Misdirected focus at the expense of appropriate measures

All participants expressed that a risk with the VZ is that healthcare will focus on the wrong things, e.g. too much focus on suicide-risk assessment at the expense of other potentially therapeutic measures, or failing to meet needs other than suicidality. More generally the participants emphasized that unachievable goals are nonconstructive, because they increase the risk of unnecessary or ineffective measures being taken at the cost of more appropriate ones.

*And many times people ask questions about suicidality even when it is not relevant, where you force the patient into, to talk about it even though you know it may be counterproductive in some cases. Not because it might induce more thoughts of suicide but because it means focusing on the wrong things.*

*The idea that if we aim for the stars we will reach the moon, no, then we might just as well, choose to aim for the moon. That will probably make it easier for us to choose considerably better means to reach the moon. When they carried out the Apollo project, no way that they were planning to go to Alpha Centauri.*

##### Threat of suicide as an instrument of power

Many participants described a tendency among some patients to use suicide threats to exert power, to get something they want, e.g. inpatient care or drugs, and that the VZ may worsen this tendency by reinforcing the threat of suicide as a weapon of negotiation.

*I can’t work as a suicide police […] if my main focus is that no one can commit suicide then, I for one become a very neurotic doctor. And then the focus will be wrong. We will fill up our wards with people who, not necessarily are the most ill ones but those who I believe will hurt themselves or commit suicide.*

##### Decreased inclination to seek help or share problems

Many participants expressed that an increased focus on suicide may have a negative effect on some patients’ inclination to seek help because of fear of compulsory care.

*What happens to their inclination to seek help the next time it’s bad, if you don’t respect that they have a wish, within what’s reasonable, to actually be able to go home with some degree of suicide risk. What happens then? They will not seek help and if they seek help they won’t tell the truth.*

#### Negative consequences for psychiatry

This category represents different ways in which the participants consider the VZ to have potentially negative consequences for psychiatry.

##### The VZ supports an unjustified difference between psychiatry and somatic care

A majority of the participants considered it a problem that the VZ contributes to what they considered an unjustified expectation that psychiatry should differ from the rest of healthcare by a responsibility to prevent every death, since no comparable goal exists in other parts of healthcare.

*And I think there is a huge fuss every time someone dies in psychiatry. If you look at the history of [a patient’s] illness you might very well think that it was amazing that this patient survived this long despite this severe mental illness and [You would like to hear] ‘what a great job you’ve done helping the patient this long’ instead of ‘no no no, what kind of terrible ward are you to let this patient die?’ I mean you accept that people die from somatic illnesses but you don’t accept that people die from, from mental illnesses.*

##### The VZ may have a deterring effect on physicians

Several participants argued that the VZ promotes the idea that every suicide is a failure on the part of healthcare and that this will discourage physicians from working in psychiatry.

*The part of the Vision Zero that implies that every suicide is a mistake and, I think this scares people away from psychiatry, because no one is prepared to take responsibility for saving, saving every one of the most unsavable persons and always live with this terror that if someone, erratically – or because it is impossible to prevent them from [doing it] – takes his life, you will have to live with that for the rest of your life, almost as if you had broken the law like a criminal. So I think it may have a discouraging effect […] I don’t think this contributes to attracting the talented [colleagues] ones so to speak. No one is prepared to sign a contract that says that I will manage preventing everything, always.*

##### The VZ may amplify contradictory tasks

Many participants perceived a contradiction regarding preventing suicides on the one hand and minimizing the use of coercive measures on the other. The VZ was perceived as worsening this problem by indicating that there are no excuses for suicides in healthcare, an interpretation that the participants felt was supported by the *Lex Maria legislation*, as it was written at the time of the interviews.

*If psychiatry in a consistent way tries to save these people’s lives, then psychiatry gets exposed in the media because of its inhumane methods. So I think it’s better to, because I mean no one wants to tie people up and so on…you would really like to just let them out. But then you’re afraid that then they will commit suicide and then the shit hits the fan. So it becomes, it’s a contradictory task when on the one hand you’re, you’re forced to stop a person from doing a certain thing, but by coercing them on the other hand you bring dishonor to psychiatry.*

##### The VZ may augment the anxiety among personnel

All participants expressed that fear of suicide is a major issue in working as a psychiatrist. Several participants expressed that the VZ has made this fear greater and that too much fear of suicide is not constructive but rather stands in the way of good healthcare.

*In psychiatry it means that you coercively detain people and deprive them of their freedom. Not because it’s the wisest measure or ethical or what you believe is the most ethically correct measure but for one reason only, and that is so that you yourself won’t have to bear the anxiety that someone may take their life […] Particularly this question of guilt, which this Vision Zero signifies is like adding fuel to this kind of behavior, that you admit [a patient] to coercive care just in case.*

*I know that several of my specialist colleagues who experienced [a patient’s] suicide, have said that ‘well, if I have to experience this again then, then I’ll stop working as a psychiatrist and change to something completely different’ And some of them got symptoms of PTSD, went to therapy, I mean people felt really, really bad […] I reached the conclusion that what it takes then is that we admit everyone who has a risk of suicide to coercive care, regardless of what they want, and then we never let them out.*

#### Wrong message

This category represents different ways in which the participants consider the VZ to be sending the wrong message to the public, to healthcare, and to patients.

##### The VZ may contribute to false views of psychiatric illness among the public

Several participants expressed that the VZ may negatively influence the public understanding of psychiatric illness by implying that psychiatric illnesses are not real illnesses by saying that every suicide is preventable. The VZ was also thought to potentially increase stigmatization by implying that psychiatric patients are in some way more responsible for their own suffering than somatic patients.

*It’s still not the case that mental illness, everywhere, is considered an illness, but that it, there might be something wrong with society or that it is a sociological concern and that, if someone only had been kind enough or if someone had helped, things surely would’ve been different. This is something I can react strongly against and, I think this plays a big role in the general stigmatization of psychiatry and mental illness, that it’s imagined not to be a real illness.*

##### The VZ may contribute to false hopes on healthcare among the public

Many participants expressed that the VZ may contribute to too high expectations of what healthcare can provide in terms of suicide prevention and treatment of psychiatric illnesses.

*I think there are many people who, for different reasons, want [to promote] a little too romantic idea of how good our chances are of making assessments and so to speak how safe the system is.*

*On the whole it is a problem, both internally and externally, when psychiatry promises and claims to be able to deliver things that it cannot deliver.*

##### The VZ contributes to medicalization of normal psychology

Some participants expressed a fear that the VZ will contribute to medicalization of normal psychological functioning and existential thinking, by implying that thoughts of suicide are inherently pathological.

*We medicalize normal reactions to life.*

*If you are a human being with an ability to reason and reflect, then you have a risk of suicide […] If we’re going to abolish suicide, one hundred percent, so that it is forbidden, then you almost have to abolish humanity, you have to lobotomize everything, everyone.*

##### Thoughts and plans of suicide as comfort and hope

Several participants conveyed that thoughts of suicide can be a source of both comfort and hope for many patients. Some participants reflected that the VZ signals that suicidal thoughts are always undesirable and that this might affect patients negatively.

*Many people live with a chronic nearness to suicide. There are those who have a rope hanging at home which has hung there for ten years and without that rope they can’t live. They know that they can go in there and for a lot of people this has a purpose - I don’t have to live, I will keep on struggling but I know I have a way out - If you removed that way out, if you could, I don’t know what would happen to a number of them.*

### The VZ is not desirable

This theme consists of four subcategories sorted under two categories and concerns what the participants perceive as reasons why the VZ is not desirable even in theory.

#### Some suicides should not be prevented because the moral cost is too high

This category represents different ways in which the participants consider the VZ, except from potentially having negative consequences, to be in direct conflict with other important values.

##### Conflicting values with regard to the patient

Many participants expressed that a problem with the VZ is that in some situations, preventing suicide is less justified, for example, when healthcare is unsuccessful in alleviating the patient’s symptoms and further measures, such as compulsory care or medication, risk inflicting more suffering.

*The more treatment-refractory a person turns out to be, I mean, the more years that have passed of compulsory care and medication and all possible things without [the patient] getting better, it only gets worse, the more I think you should question if it’s actually meaningful. Because it has no intrinsic value, but it should aim for making the patient feel better and getting well, and if it doesn’t it only does damage.*

##### Conflicting values with regard to others

Many participants give examples of the VZ being in conflict with the interests of other people. Examples evolve around children’s rights, family policy, surveillance, alcohol restriction, and situations where society has an interest in applying legal measures, e.g. legal sentences, economic penalties, and eviction, that are associated with an increased risk of suicide.

*There was always a discussion if you should, so to speak, take [the patient’s] her child away or not. Because then you knew she would attempt suicide immediately. In such situations [the patient] she managed to disarm the whole system that aspired to protect the child. Because no one wanted to press the death-button so to speak, so the child did fare ill in that way and probably still does.*

*To what extent should you be allowed to fail in life […] If you’re sentenced for a crime, the suicide risk also goes up sky high the following months. If you go bankrupt same thing, the suicide risk goes up sky high. If you’re evicted from your home, same thing. So there are several examples of failures or shameful situations in life that we know to be associated with a high risk of suicide. Then of course it would be possible to simply stop sentencing people for a lot of crimes.*

#### Certain rational suicides should not be prevented

This category represents different ways in which the participants considered the VZ to not be desirable in theory, by arguing that certain rational suicides should not be prevented.

##### Rational suicide

Many participants spontaneously brought up what they perceived as rational suicides, without being asked about this. Rational suicide was used as an argument for why the VZ is not a desirable goal.

*In some way it [the VZ] implies that every suicide is wrong even though many people suggest that there is the rational suicide. And there are also those [cases] that may be somewhat rational as well as having elements of psychiatric illness but not to the extent that you could prevent them anyway. And this is something that, it’s political suicide, interestingly enough, should one say that in public, that we have to accept certain suicides.*

##### Assisted suicide

The participants expressed both the idea that legalization of assisted suicide could have a suicide preventive effect, as well as the opposite idea that it would rather be a way of facilitating suicide.

*There are also people who should be allowed to take their own life […] different people with ALS and Huntington and things like that. Then it’s obvious, it’s totally, it’s a shame for this country that it’s not legal for people like that to be able to get euthanasia.*

## Discussion

Though most of the participants mentioned at least some potential benefit of the Vision Zero for Suicide, the overall impression was a predominant skepticism towards it. Some participants focused on why they consider the VZ to be unachievable, while others focused more on its potential consequences and normative implications. Accordingly, the analysis of the interviews yielded themes that reflect these different focuses. An overarching impression is that the participants shared the belief that the number of suicides should and could be reduced, but that the VZ nonetheless, for different reasons, is not desirable.

### Unachievable, nonconstructive or counterproductive

All participants considered the VZ to be unachievable. The major reason was a lack of reliable methods for suicide risk assessment. This is consistent with a recent report from the *Swedish agency for health technology assessment and assessment of social services* (SBU), *“*Instruments for Suicide Risk Assessment” from 2015, which concludes that “none of the included studies provided scientific evidence to support that any instrument had sufficient accuracy to predict future suicide with 80% sensitivity and 50% specificity” [32]. The report supports the participants’ notion that in order to reduce the numbers of suicides, the methods for prediction of suicide need to be substantially improved.

The VZ was considered to be unachievable also because of limitations of contemporary psychiatry: current clinical methods are not effective enough to save every person at risk of suicide. This, too, is consistent with the existing literature [33]. A related concern was that the VZ will contribute to the general public underestimating the severity of psychiatric illnesses or overestimating the ability of healthcare to treat these illnesses. These shortcomings may then lead to decreased public trust in psychiatry.

Some participants expressed a fear that increased focus on suicide may have problematic clinical effects or may even be counterproductive to its own purpose. First, it may decrease some patients’ inclination to seek help because of fear of compulsory care. Second, it may decrease some patients’ trust in healthcare by signaling that only suicidality is important. Third, a focus on suicidality at the expense of other psychiatric problems may for some patients lead to suicidality later on, without guarantees that they will seek help when they eventually are suicidal. Fourth, it may put the psychiatrist in a weakened position since it strengthens the patient’s chances of using suicidal behavior as a weapon of negotiation. Fifth, an increased focus on suicidality does not necessarily mean better suicide prevention. Some participants expressed that the current implementation of suicide prevention in healthcare places too much emphasis on mandatory suicide risk assessments and that this is not an effective way of preventing suicide. Moreover, they expressed that mandatory risk assessments take time from other potentially therapeutic activities, may have a negative impact on the psychiatric consultation and may disturb the patient-physician relationship by thwarting the consultation. Beyond these concerns, it may be added that even if not counterproductive, it is a normative question whether prioritizing suicide risk over other psychiatric problems in general is ethically justifiable.

A major impression from the interviews is that there was a great deal of anxiety among the interviewed psychiatrists that patients will complete suicide. The participants reported considerable psychological suffering and morbidity among personnel facing patients’ suicides; they felt that the VZ has worsened these anxieties. Several participants perceived the task of trying to limit the use of compulsory care and at the same time preventing every potential suicide as contradictory; the lack of clear directives on how to balance these tasks added to this frustration. Furthermore, these problems may, some participants maintained, deter people from working with psychiatry and invite resignation in others. This result is consistent with points raised in the debate in the Swedish medical journal, Läkartidningen, where one of the arguments against the VZ was that it places an unreasonable burden on psychiatry [23].

### Comparing psychiatry with somatic healthcare

The participants expressed that the VZ has several problematic implications for the understanding of psychiatric illness and for psychiatry. There was also a widespread sense of unfairness with regard to the Swedish *Lex Maria legislation* [11]. This legislation originally addressed critical incidents caused by medical errors, and regulations from the Swedish National Board of Health and Welfare – to which the VZ is linked – stated that all instances of suicide in relation to healthcare should be investigated accordingly. As mentioned these regulations are now changed. Nevertheless, the participants perceived the VZ as a liability mainly because it was perceived to imply that every suicide is a failure on the part of healthcare. This problem was elaborated by the participants in three principal ways: (1) it implies that psychiatry could successfully save the life of all of its patients if certain measures are taken, in contrast to somatic healthcare, in which a certain amount of mortality is accepted with reference to current limitations of medicine; (2) in a contradictory way it implies a difference between psychiatric and somatic illness: psychiatric patients are *less* responsible for their own wellbeing since suicide is considered a failure on the part of healthcare, and at the same time *more* responsible since they are assumed to be able to *choose* not to complete suicide, given that psychiatry acts in a certain way; (3) it implies that suicidal ideation is always pathological. Some participants expressed a concern that the VZ will contribute to the medicalization of normal human psychology as well as to increased shame and stigmatization of suicidal patients. These results echoed the debate in Läkartidningen, in which one argument against the VZ has been that it will increase shame and stigmatization of suicidal persons by implying that thoughts of suicide are always wrong. Again, this is a way by which the VZ is thought to be potentially counterproductive; the option of suicide itself is sometimes thought to be protective against suicide. It is also noteworthy that the participants again alluded to a major discussion in the bioethical literature, namely the medicalization of normal human psychology [34].

Although a huge area in modern biomedical ethics [35], a further discussion of whether or not psychiatric and somatic illnesses are equal in all relevant respects is beyond the scope of this paper. It is important to note, however, that if healthcare treats psychiatric and somatic patients differently, and if there is a difference in the responsibility of healthcare professionals for mortality in the respective groups, arguments are needed to justify these differences.

### Undesirable because of ethical conflicts

Several participants expressed arguments why the VZ is not desirable because it conflicts with other important values. First of all, they emphasized that measures aimed at treatment or suicide prevention, e.g. compulsory care and medication, sometimes inflict further suffering on the patient. They also emphasized that inpatient care is the only way for the responsible physician to be fairly certain that a patient will not attempt suicide, at least in the short term. However, they also expressed that the element of coercion itself often lowers the chances of therapeutic success and, further, that the level of unnecessary coercion is considerable and that it is fairly common to overuse coercive care ‘just in case’, in fear of the patient attempting suicide. The VZ was thought to inappropriately amplify these tendencies by increasing the focus on suicide.

One way of justifying measures that are potentially harmful is by reference to the aim of decreasing future suffering or saving or prolonging life. Thus, the more unlikely the wanted outcome is (prolonged life or decreased future suffering), the weaker the justification. The claims that psychiatry is not successful in treating all its patients and that compulsory care can be potentially harmful are fairly uncontroversial. Previous research indicates that suicide risk is perhaps the most important issue for justifying compulsory psychiatric care [36], explaining to some degree the participants’ concern that the VZ may increase the use of coercion in healthcare. However, the question whether the VZ actually does so is an empirical one yet to be answered. Further empirical questions yet to be answered are whether compulsory care on current rates in fact lowers the suicide rates, and whether increasing the use of compulsory care would be counterproductive to suicide prevention. These uncertainties pose challenging problems for answering ethical questions regarding the justification of compulsory care as a method of suicide prevention. Suffice it to note that these considerations again point towards the question of what means are justified in order to achieve suicide prevention. It is also interesting to note that these claims are consistent with the cited debate in Läkartidningen, where one argument against the VZ has been that it may be counterproductive by increasing the use of compulsory care. The results in this study shed some light on why the VZ could have such an effect [23].

A different argument why the VZ is not desirable given by some participants was that it may be in conflict with the interests of the general public, e.g. in the areas of children’s rights, family policy, surveillance, alcohol policy, and in instances where society has an interest in applying legal measures associated with increased risk of suicide, such as legal verdicts, economic penalties, and eviction. This argument again points towards the question of what means are justified to achieve suicide prevention, but this time alludes to values other than those of the patient.

The issue of rational suicide was discussed extensively by the participants, giving rise to several interesting questions. It is the authors’ ambition to present the results from these discussions in a separate article. Here, with respect to the aim of this article, only a brief account of these results will be made: Several participants recognized the possibility of rational suicides, typically exemplified by cases in which an individual suffers from a severe somatic illness without hope of improvement and associated with considerable suffering. Some participants expressed that severe, chronic psychiatric illness, too, could serve as a rationale for suicide. The participants conveyed that what makes these questions difficult is not so much the question of whether rational suicides exist, but rather that there is a considerable “grey zone” in which the wish to die can be considered partly rational and partly irrational. Further, that it is either impossible or extremely difficult to assess the rationality of a patient’s suicidal thoughts. One possible interpretation of these statements is that the participants tended to consider the possible existence of rational suicides as less relevant from a clinical perspective; the vast majority of suicidal patients are considered irrational until the opposite can be proven. These results are in line with previous research on psychiatrists’ views on rational suicide [36].

Importantly, the approach of presupposing irrationality is problematic since suicide prevention then comes into potential conflict with respect for autonomy – as is the case when suicidal patients are considered irrational, and thereby non-autonomous, “by default”, i.e., without prior consideration. Respect for autonomy is considered very important in healthcare legislations in most Western countries. An important question arises: does the VZ protect important values to the extent that disregarding autonomy in these cases is justifiable?

### Strengths and weaknesses

This study includes a relatively large number of interviews with psychiatrists who differ in age, experience and area of expertise. The result is rich and varied, which was also the aim of the method, and we consider this to be one of the strengths of the study. Nonetheless the applied chain referral recruitment of participants may have led to a selection of participants with similar perceptions of the Vision Zero for Suicide. However, the richness and variation of the material speaks against that this would be the case. The results cannot be used to infer any general conclusions on how common certain perceptions and arguments are among Swedish psychiatrists, and there is a possibility that more interviews would have yielded yet other understandings and arguments not found in this material. However, since the aim of the study was to enrich the normative discussion on the Vision Zero for Suicide by examining perceptions and arguments among Swedish psychiatrists, the results constitute an empirical knowledge base. If the normative discussion can be improved by these results, it may be relevant also for other countries in setting goals for suicide prevention.

## Conclusions

Though most of the participants mentioned at least some potential benefit of the Vision Zero for Suicide, there was a predominant skepticism towards it. The VZ was perceived to be impossible to realize, nonconstructive or potentially counterproductive, as well as undesirable because of potential conflicts with other values and interests of both patients and the general public. Contradictory tasks and unclear directives on what means are morally justified in suicide prevention were held to be especially troubling concerns by the participants. Moreover, the VZ appears to be perceived as having negative consequences for the working conditions for psychiatrists in Sweden, by increasing psychiatrists’ anxiety and thwarting the patient-physician relationship.

## Additional file

Additional file 1: Interview guide containing questions about the participants’ experiences and perceptions of the Vision Zero for Suicide as well as questions about rational suicides. (DOCX 15 kb)

## Abbreviations

VZ: Vision Zero for Suicide

## References

[1] World Health Organization (2014). WHO | preventing suicide: a global imperative.

[2] Självmord i Sverige | Nationellt centrum för suicidforskning och prevention av psykisk ohälsa | Karolinska Institutet. [cited 2016 Mar 4]. Available from: http://ki.se/nasp/sjalvmord-i-sverige-0.

[3] WHO | Suicide. WHO. World Health Organization; 2018 [cited 2018 Feb 1]. Available from: http://www.who.int/mediacentre/factsheets/fs398/en/.

[4] Titelman D, Oskarsson H, Wahlbeck K, Nordentoft M, Mehlum L, Jiang G-X (2013). Suicide mortality trends in the Nordic countries 1980-2009. Nord J Psychiatry.

[5] Socialstyrelsen. Statistics on Causes of Death 2016. Statistics on Causes of Death 2016. 2017 [cited 2018 Feb 7]. Available from: http://www.socialstyrelsen.se/publikationer2017/2017-9-11.

[6] Zero Suicide. [cited 2018 Feb 6]. Available from: http://zerosuicide.sprc.org/.

[7] (US) O of the SG, (US) NAA for SP. 2012 National Strategy for suicide prevention: goals and objectives for action. 2012 National Strategy for suicide prevention: goals and objectives for action: a report of the U.S. surgeon general and of the National Action Alliance for suicide prevention. US Department of Health & human services (US); 2012 [cited 2018 Feb 1]. Available from: http://www.ncbi.nlm.nih.gov/pubmed/23136686. 23136686

[8] Socialdepartementet. Prop. 2007/08:110 - En förnyad folkhälsopolitik. Stockholm; 2008. Available from: http://www.regeringen.se/sb/d/9251/a/100978.

[9] Socialstyrelsen. HSLF-FS 2017:41 Inspektionen för vård och omsorgs föreskrifter om anmälan av händelser som har medfört eller hade kunnat medföra en allvarlig vårdskada (lex Maria) [Internet]. ISSN 2002–1054, Artikelnummer IVO 2017–14 Sweden; 2017. Available from: https://www.ivo.se/publicerat-material/foreskrifter/hslf-fs-2017-41/.

[10] Socialstyrelsen. Socialstyrelsens föreskrifter och allmänna råd (HSLF-FS 2017:40) om vårdgivares systematiska patientsäkerhetsarbete. ISSN 2002-1054, Artikelnummer 2017-5-24 Sweden; 2017. Available from: https://patientsakerhet.socialstyrelsen.se/om-patientsakerhet/centrala-lagar-och-foreskrifter/hslf-fs-2017-40.

[11] Socialstyrelsen. SOSFS 2005:28. Socialstyrelsens föreskrifter och allmänna råd om anmälningsskyldighet enligt Lex Maria.

[12] Albinsson A, Willems B (2011). Socialstyrelsens kritik bygger på teorier och önsketänkande. Lakartidningen.

[13] Holm H (2009). Nollvision skapar nya tabun. Lakartidningen.

[14] Sunesson P-A, Tegnell A (2011). Granskningar gör vården säkrare. Lakartidningen.

[15] Wasserman D, Beskow J, Jakobsson L, Spångberg B, Falkenroth A, Hägglöf B (2009). Nollvisionen ger viktiga signaler. Lakartidningen.

[16] Westerhäll LV (2011). Psyskiatrisk tvångsvård kontra nollvision om suicid. Lakartidningen.

[17] Albinsson A, Willems B (2011). Slutreplik om suicidprevention: Den dystra sanningen är att ingen vet hur man gör. Lakartidningen.

[18] Wasserman D, Stain R, Carli V (2013). Fördjupat kunskapsunderlag för att förhindra självmord. Lakartidningen.

[19] Bergmark T (2009). Motsägelsefullt om nollvisionen för självmord. Läkartidningen nr 20 volym.

[20] Battin MP. Suicide. In: S.G. Post, editor in chief. Encyclopedia of bioethics, 3rd edition. New York: Macmillan. 2003. p. 2475–2483.

[21] Cavanagh JTO, Carson AJ, Sharpe M, Lawrie SM (2003). Psychological autopsy studies of suicide: a systematic review. Psychol Med.

[22] Radhakrishnan R, Andrade C (2012). Suicide: an Indian perspective. Indian J Psychiatry.

[23] Holm H, Sahlin N-E (2009). Regeringens nollvision för självmord kan få motsatt effekt. Lakartidningen.

[24] Tännsjö T (1999). Coercive care: the ethics of choice in health and medicine.

[25] Patton MQ. Qualitative Research & Evaluation Methods. Fourth edition. Los Angeles: SAGE Publications, Inc; 2015.

[26] Tansey O (2007). Process A Case Tracing and Elite interviewing : sampling for non-probability process tracing : definition. PS Polit Sci Polit.

[27] Sandelowski M, Sandelowski M (2000). Focus on research methods whatever happened to qualitative description?. Res Nurs Health.

[28] Green J, Thorogood N. Qualitative methods for Health Research. Second edition. London: SAGE Publications Ltd; 2009.

[29] Kvale S, Brinkmann S (2014). Den Kvalitativa Forskningsintervjun.

[30] Meadows KA (2003). So you want to do research? 3. An introduction to qualitative methods. Br J Community Nurs.

[31] Graneheim UH, Lundman B (2004). Qualitative content analysis in nursing research: concepts, procedures and measures to achieve trustworthiness. Nurse Educ Today.

[32] SBU (2015). Instrument för bedömning av suicidrisk. En systematisk litteraturöversikt.

[33] Sadock BJ (2012). Inevitable suicide: a new paradigm in psychiatry. J Psychiatr Pract.

[34] Sedler MJ (2015). Medicalization in psychiatry: the medical model, descriptive diagnosis, and lost knowledge. Med Health Care Philos.

[35] Hope T (2004). Medical ethics: a very short introduction.

[36] Sjöstrand M, Sandman L, Karlsson P, Helgesson G, Eriksson S, Juth N (2015). Ethical deliberations about involuntary treatment: interviews with Swedish psychiatrists. BMC Med Ethics.

