# Supplementary material for: Skepticism towards the Swedish vision zero for suicide: interviews with 12 psychiatrists
Source: BMC Med Ethics. 2018 Apr 10;19:26. doi: 10.1186/s12910-018-0265-6 (PMC5894210; doi:10.1186/s12910-018-0265-6)
Supplement: Supplementary file 1 — Interview guide containing questions about the participants’ experiences and perceptions of the Vision Zero for Suicide as well as questions about rational suicides. (DOCX 15 kb) [file 12910_2018_265_MOESM1_ESM.docx]

**Interview guide**

1. What is your background and clinical experience?
2. Are you familiar with the Vision Zero for Suicide? How do you interpret it?
3. Has the Vision Zero for Suicide affected your clinical work?
   1. In that case, how?
   2. Do you think that Swedish health care/psychiatry in general has been affected by the Vision Zero for Suicide?
4. What do you think about the Vision Zero for Suicide?
   1. What is positive about it?
   2. What is negative about it?
5. Do you believe that all suicides can be explained by psychiatric illness?
   1. What does the concept “rational suicide” mean to you?
   2. If rational suicides exist, is it possible for a third party/a psychiatrist to assess whether the intention is rational?
   3. If rational suicides exist, should they also be prevented?
   4. Is it conceivable that a person would wish to end his/her own life because of severe psychiatric suffering and that this wish would be rational?
